# Insulin Resistance and NAFLD: A Dangerous Liaison beyond the Genetics

**DOI:** 10.3390/children4080074

**Published:** 2017-08-14

**Authors:** Melania Manco

**Affiliations:** Bambino Gesù Children’s Hospital, Research Unit for Multifactorial Diseases, Via Ferdinando Baldelli 38, 00146 Rome, Italy; melania.manco@opbg.net; Tel.: +39-066-859-2649

**Keywords:** insulin resistance, non-alcoholic fatty liver diseases, obesity

## Abstract

Over the last decade, the understanding of the association between insulin resistance (IR) and non-alcoholic fatty liver disease (NAFLD) has dramatically evolved. There is clear understanding that carriers of some common genetic variants, i.e., the patatin-like phospholipase domain-containing 3 (PNPLA3) or the transmembrane 6 superfamily member 2 (TM6SF2) are at risk of developing severe forms of NAFLD even in the presence of reduced or absent IR. In contrast, there are obese patients with “metabolic” (non-genetically driven) NAFLD who present severe IR. Owing to the epidemic obesity and the high prevalence of these genetic variants in the general population, the number of pediatric cases with combination of genetic and metabolic NAFLD is expected to be very high. Gut dysbiosis, excessive dietary intake of saturated fats/fructose-enriched foods and exposure to some chemicals contribute all to both IR and NAFLD, adding further complexity to the understanding of their relationship. Once NAFLD is established, IR can accelerate the progression to the more severe form of liver derangement that is the non-alcoholic steatohepatitis.

## 1. Introduction

Insulin resistance (IR) is one of the hallmarks of non-alcoholic fatty liver disease (NAFLD) being pivotal in the pathogenesis of the disease as associated to obesity [1]. IR is, moreover, one of the multiple hits determining the progression from NAFLD to non-alcoholic steatohepatitis (NASH) [2].

In the late 1990s, Marchesini et al. [3] first pointed out the significant association between NAFLD and IR, demonstrating that adult patients with NAFLD have insulin sensitivity and hepatic glucose production (HGP) as impaired as patients with overt type 2 diabetes (T2D). The reduced insulin sensitivity and the inappropriate HGP of the NAFLD patients were irrespective of their body fatness and glucose tolerance [4]. In 2003, Jeffrey Schwimmer et al. [5] demonstrated severe IR as estimated by the homeostasis model assessment of insulin resistance (HOMA-IR) in 43 children and adolescents with NAFLD. Six of them had T2D. Mean value of HOMA-IR was five to six fold-increased respect to values reported as normal in normal-weight healthy children and adolescents [6]. In the Schwimmer’s series of NAFLD young patients, steatosis was predicted by the combination of the quantitative insulin sensitivity check index (QUICKI), age, and ethnicity [5].

After this first report, the association between NAFLD and IR was replicated in a number of pediatric case-series of overweight and obese patients.

The main reason for the almost constant triad among obesity–IR–NAFLD relates to some defects of the fat partitioning that impair the cross talk between the liver and the adipose tissue and cause the overflow of lipids to the liver and/or their defective export from the organ.

The first intention of this review is to recap basic concepts about systemic (_S_IR) and hepatic insulin resistance (_H_IR) and illustrate how impaired fat partitioning affects IR in patients with fatty liver. Since some defects of lipid partitioning are genetically driven, endophenotypic differences between pure “metabolic” and “genetic” NAFLD will be discussed. A mouse model that mimics fatty liver deriving from increased hepatic de novo lipogenesis (DNL) or increased adipose tissue lipolysis will be described as being explanatory of some pathogenic mechanisms that are unique to the pure metabolic or the pure genetic form. In true life, these pathogenic mechanisms mix up and result in a variety of phenotypes ranging from mild NAFLD with no IR to combination of both severe NAFLD and IR.

IR refers to a defective metabolic response to the effect of the hormone in the target cell (i.e., muscle cell, hepatocyte, adipocyte, etc.) or at whole organism level. For the purpose of this review, we will discuss systemic and hepatic IR [7].

_S_IR indicates the inability of insulin to lower appropriately blood glucose levels owing to the disrupted translocation of the GLUT4 receptor to the surface membrane of the muscle cell that results in reduced insulin dependent uptake of glucose [7]. The uptake of glucose can be quantified by using the euglycemic hyperinsulinemic clamp (EHC) that is the gold standard to estimate the systemic insulin sensitivity [8]. Since the technique is unpractical in clinical practice and large population studies, surrogate indexes have been adopted. The HOMA-IR [9] is the most frequently used index of IR among those based on fasting value of glucose and insulin, while the QUICKI [10] corresponds to 1/HOMA-IR and such as is an index of insulin sensitivity. The Matsuda’s insulin sensitivity index [11] and the Mari’s oral glucose insulin sensitivity [12] are calculated upon values of glucose and insulin during the oral glucose tolerance test (OGTT).

_H_IR consists of disturbed insulin mediated suppression of hepatic glucose production (HGP) but in presence of preserved stimulation of lipogenesis [7]. The gold standard method for measuring _H_IR is a tracer dilution study incorporating a stepped clamp [7]. Again, this kind of investigation is not feasible in larger metabolic studies and would not be suitable for routine clinical use. HOMA-IR, which is based upon the correlation between fasting insulin and glucose values, is related to _H_IR more than to _S_IR and is deemed as a reliable index of _H_IR [13].

In the adipose tissue, insulin resistance (_AT_IR) means the ineffective suppression of lipolysis by the hormone.

## 2. Insulin Resistance, Disrupted Fat Partitioning and Hepatic Steatosis

Mechanisms of the association between IR and NAFLD have been widely investigated but recent insight about the genetics and the natural history of the disease has provided a better understanding of their relationship [14].

Obese children and adolescents with NAFLD present generally with both _H_IR and _S_IR [15], but in early-onset NAFLD, neither _H_IR nor _S_IR were found [16]. NAFLD was suspected in some preschoolers (age 2–6 years) with recent onset of excess weight. Suspicions were based on enhanced liver brightness and altered liver function tests in absence of any other reasonable cause. In these cases, HOMA-IR and Matsuda index were not different from median values of BMI and age matched patients with no suspicious [16].

**Figure 1:**
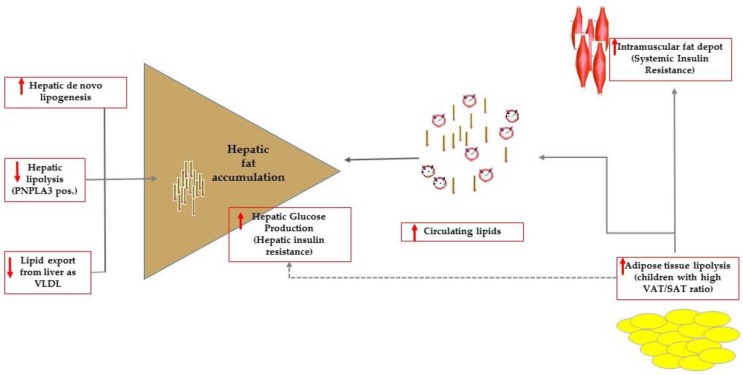
Summary of the mechanisms favoring intrahepatic accumulation of lipids that are, namely: increased de novo lipogenesis, reduced hepatic lipolysis (this mechanism can be effective in carriers of the PNPLA3 variant) and export as very low dense lipoproteins (VLDL). In patients with high visceral adipose (VAT) to subcutaneous (SAT) tissue ratio, impaired adipose tissue lipolysis caused overflow of lipids to ectopic tissues including the muscle tissue. At the level of the muscle tissue, fat accumulation results ultimately in systemic insulin resistance. Adipose tissue lipolysis contributes to control hepatic glucose production that is enhanced in patients with hepatic steatosis who have commonly impaired adipose tissue lipolysis.

These findings suggest that both hepatic and systemic IR would develop later. This notion is in keeping with the Danforth’s [17] hypothesis, then revised by Vidal-Puig [18]. Researchers have speculated about an inadequate subcutaneous adipose tissue (SAT), unable to expand, as a culprit for excessive lipid flux to the visceral adipose tissue (VAT) and to non-adipose tissues [17,18]. Elevated hepatic fat deposition results from the imbalance among lipid synthesis (hepatic DNL); uptake and esterification of fatty acids from sources such as adipose tissue lipolysis and dietary fats; and hepatic lipid export in the form of triglyceride-rich VLDL (Figure 1). While hepatic DNL does not necessarily lead to IR [19], _S_IR and _H_IR are likely to develop when availability of lipids exceeds the lipid accumulation capacity in humans [20].

In children and adolescents whose visceral adipose tissue becomes insufficient to store excess fats, the latter overflow to the visceral compartment and start to accumulate ectopically in key sensitive organs, i.e., muscle tissue and liver, hence causing IR. As elegantly revised in a recent review paper [21], in children and adolescents with “metabolic” NAFLD the assumption that the overflow of lipids to the liver is a consequence of the inadequate storage of excessive fat in the subcutaneous depot remains valid. These obese youngsters with NAFLD have a distinct endophenotype characterized by a thin superficial layer of SAT, increased VAT, marked _S_IR, _H_IR, and dyslipidemia as compared to age and BMI matched patients with no NAFLD [22]. IR worsens progressively as the VAT to SAT ratio increases and the hepatic steatosis becomes the most important marker of IR, glucose dysregulation and cardiometabolic risk [23]. At biopsy, adipocytes from these patients present with coexistence of large and small adipocytes as opposed to a more homogeneous cellularity in obese patients with low VAT to SAT ratio. Adipocytes also show macrophage infiltration and down regulation of key lipogenic/adipogenic genes and sirtuin 1_a protein known to modulate the cell response to stress and apoptosis [23]. In a different study, patients with similar defective lipid partitioning also had impaired insulin suppression of lipolysis [22]. It is worth mentioning that impaired lipolysis is particularly important to explain the significant association between NAFLD and risk of T2D. In the physiological condition, insulin controls HGP by regulating adipocyte lipolysis [24] and thus reducing fatty acid flux to the liver. This results in the reduced availability of hepatic acetyl coenzyme A (CoA) concentrations and decreased pyruvate carboxylase activity with consequently decreased conversion of pyruvate to glucose. In obese patients with NAFLD, the impaired lipolysis causes inappropriately enhanced HGP.

## 3. Intrahepatic Fat Quality More than Quantity Impacts on Insulin Resistance

NAFLD develops as the consequence of acquired impaired lipid partitioning (“metabolic” NAFLD) but genetics can play an important role in the onset and/or progression of the disease as reviewed in this issue of the Journal by Umano et al. [25]. A number of gene variants have been associated with increased risk of NAFLD and the association of the patatin-like phospholipase domain-containing 3 (PNPLA3) and the transmembrane 6 superfamily member 2 (TM6SF2) have been robustly replicated in children too [26,27,28,29]. While conferring an increased risk for NAFLD, the I148M variant in PNPLA3 and/or the E167K variant in TM6SF2 are not associated with increased risk for IR and any associated metabolic abnormality including T2D [19,26]. In a meta-analysis [29], carriers of the I148M variant had 73% more liver fat than non-carriers. Insulin resistance/sensitivity as evaluated by HOMA-IR, EHC, fasting or post-glucose insulin and glucose concentrations did not differ between carriers and non-carriers of the gene variant. Studies included obese and non-obese, diabetic and non-diabetic as well as pediatric cohorts. Serum triglycerides were either similar or lower in variant allele carriers as compared to non-carriers, consistent with lack of IR.

In vitro, the PNPLA3 I148M gene variant abolishes intra-hepatocellular lipolysis [30,31] and by acting as a lysophosphatidic acid acyl transferase stimulates triglyceride synthesis from long unsaturated fatty acids containing coenzyme A (CoA) more than from saturated fatty acid CoAs, hence resulting in increased DNL [32]. As it has been stated above, hepatic DNL does not necessarily cause IR [19]. The length of chains and the lipid structure of fats accumulating within the parenchyma are likely to make the difference. Indeed, studies proved that carriers of the PNPLA3 I148M variant had a different intrahepatic lipid profile with respect to non-carriers [33]. Polyunsaturated triglycerides were abundant in the liver fat of the PNPLA3 I148M gene variant carriers, whereas in “metabolic” NAFLD the concentration of saturated triglycerides and ceramides was increased [33]. A depot of ceramides was associated to the derangement of the hepatic insulin metabolism demonstrating that the lipid profile and not the amount influences the occurrence of the _H_IR [33].

A number of studies found adults and children carrying the TM6SF2 E167K variant to have a significantly higher liver fat content than non-carriers [27,28,34,35]. Adult carriers of the variant represent approximately 7% of all subjects [35]. The polymorphism determined about a two fold-increased risk of NAFLD [35] and the risk was increased independently of the genetic variation in PNPLA3 at rs738409, obesity and alcohol intake [34]. Carriers had lower serum concentration of triglycerides, total and low-density lipoprotein (LDL) cholesterol than non-carriers [35]. Insulin sensitivity, as determined in fasting or post OGTT conditions did not differ between carriers and non-carriers [35]. The TM6SF2 variant was also genotyped in a multiethnic cohort of 957 obese children and adolescents (42% Caucasians, 30% Hispanics, 28% African Americans). Of them, 454 children underwent a magnetic resonance imaging study to assess hepatic fat content. The gene variant was associated with high hepatic fat content in Caucasians and African Americans, with high alanine aminotransferase levels in Hispanics but a more favorable lipoprotein profile (lower LDL, small dense LDL, and very small LDL) in Caucasians and Hispanics. In the few cases who underwent the liver biopsy, it showed a higher prevalence of fibrosis and nonalcoholic fatty liver disease activity score in carriers [27]. The variant was also genotyped in a sample of over 1000 Italian obese children and adolescents and carriers had more severe steatosis than non-carriers, increased levels of liver enzymes, but a better lipid profile. The study confirmed no association of NAFLD with IR [28]. Genetic differences can partly explain the reason why prevalence of IR, NAFLD and their association are different among the different ethnicities [36].

## 4. Lesson from the Animal Model

The hyperinsulinemia and the hyperglycemia that typically occur in an obese individual, promote DNL by upregulating lipogenic transcription factors, such as sterol regulatory element binding protein-1c (SREBP-1c) and carbohydrate response element binding protein (CREBP) [37,38]. Insulin-mediated activation of SREBP-1c increases malonyl-CoA, a key intermediate of the fatty acid synthesis, which inhibits carnitine palmitoyltransferase 1, long chain fatty acid entry into the mitochondria for β-oxidation and thus favors hepatic triglyceride accumulation [39,40]. A very recent study [41] was conducted in mice overexpressing hepatocyte- or adipocyte-specific SREBP-1c to dissect metabolic differences between NAFLD mainly deriving from increased hepatic *DNL* (termed as “primary” NAFLD) or owing to adipose-tissue lipolysis (“secondary” NAFLD). While primary NAFLD may resemble, even with limits, pathogenesis of the disease in carriers of genetic variants predisposing to the intrahepatic accumulation of fat, secondary NAFLD may be more like the metabolic form in humans in whom the increased lipolysis causes fat overflow to the liver and impaired HGP. Primary NAFLD mice featured increased lipogenic gene expression in liver and adipose tissue; and _H_IR that was associated with increased C18:1-diacylglycerol (DAG) content and protein kinase C (PKC)ε translocation. Mice with secondary NAFLD had decreased hepatic ChREBP-mediated lipogenesis and featured _S_IR. Results of the study suggested that _S_IR develops following increased hepatic lipogenesis only if adipose tissue lipid storage capacity is impaired, as it happens in human “metabolic” NAFLD.

## 5. The Real-Life Scenario

Gene variants that promote hepatic steatosis may be deemed as “thrifty” genes, i.e., genes which enable individuals to efficiently store energy as fat during periods of food abundance to survive feast and famine and to child-bear [42]. While intrahepatic glycogen is an energy reservoir for short-term supply of energy, intrahepatic fat serves for the long-term. Accordingly, carriers may have been favored trough centuries since they were able to survive famine periods and these variants have become common. For instance, about 30% of Europids carry the PNPLA3 I148M variant [43].

Considering such high prevalence of carriers and the obesity epidemic, many young patients will have ‘double trouble’ and might develop early onset and severe NAFLD [44]. In these patients, the progression from simple steatosis to steatohepatitis seems to be accelerated with a rate that is not dependent on the degree of IR [29]. Other factors seem to add complexity and shape phenotypes of risks along a continuum in terms of insulin resistance, cardiovascular risk and fatty liver. They include exposure to gut dysbiosis [45] and gut-derived lipopolysaccharides (LPSs) [46], nutrition imbalance with excessive dietary intake of saturated fats and fructose [47,48,49,50], and exposure to chemicals acting as endocrine disruptors [51].

In the pediatric population, metagenomic signatures with a decrease of Oscillospira, an increase of Ruminococcus and Dorea have been linked clearly to NAFLD. Nevertheless, the impact of such a dysbiosis on in vivo estimates of IR needs still to be ruled out [45]. LPSs from the gram-negative bacterial wall are the most powerful ligand of the toll like receptor 4, whose activation triggers low-grade inflammation and, in turn, impacts on non-alcoholic steatohepatitis, IR and cardiovascular disease [46].

The cafeteria diet (a diet consisting of food regularly consumed by humans, including high-salt, high-fat, low-fiber, energy dense foods such as cookies, chips, and processed meats) [52] in animal models and the westernized diet in human epidemiological studies have been proved to cause SIR. These dietary regimens have been also implied in the pathogenesis of fatty liver and progression to NASH with their high content of saturated fats and fructose [47,48,49].

Finally, children are commonly exposed to some chemicals that are able to reduce systemic insulin sensitivity while accumulating in the liver where they interfere with insulin and lipid metabolism [51]. Such an intricate scenario needs to be detangled by using a likewise complex investigative approach that integrates clinical and omics phenotypes in order to provide a better prevention and personalized therapy [53]. In this complex scenario, it is hard to identify what comes first, the NAFLD or the IR since they recognize the same pathogenic factors.

## 6. Conclusions

Recent understanding of the genetic origin of NAFLD can help to explain why the interplay between NAFLD and IR results in phenotypes of NAFLD from modest to severe accompanied by IR ranging from null to severe. Genetic differences may also explain why very young patients present with severe histological involvement of the hepatic parenchyma. Nonetheless, insulin resistance is still a pivotal player in the pathogenesis and progression of the NAFLD. A number of causative factors converge on increased IR as common soil for the development of NAFLD.
